# The Effect of High-Pressure Processing on the Copigmentation and Storage Stability of Polyphenols with Anthocyanin Monomers

**DOI:** 10.3390/foods13233756

**Published:** 2024-11-23

**Authors:** Yuxuan Sun, Fang Huang, Yan Chen, Nan Ning, Gang Hao, Xiufang Bi

**Affiliations:** College of Pharmacy and Food, Southwest Minzu University, Chengdu 610041, China; stellasun1540@gmail.com (Y.S.); huangfang1925@outlook.com (F.H.); cyan95129@163.com (Y.C.); blipu62@163.com (N.N.); indianahg@hotmail.com (G.H.)

**Keywords:** anthocyanin, high-pressure processing, polyphenols, molecular docking

## Abstract

This study aims to determine the effect of different high-pressure processing (HPP) conditions (100 MPa/300 MPa/500 MPa; 2 min/4 min/6 min) on copigmentation, specifically between chlorogenic acid (CA), epicatechin (Epi), gallic acid (GA), malvidin-3-O-galactoside (Mv-3-O-gal), and malvidin-3-O-arabinoside (Mv-3-O-ara), as well as the storage stability of the copigmentation solutions. The results showed that the influence of different HPP treatment conditions on copigmentation was not significant. HPP treatment did not significantly affect the λ_max_, peak absorption, color parameters, and Mv-3-O-gal anthocyanin content when applied alone or in combination with CA and Epi. However, the color intensity and a* value of Mv-3-O-gal with GA decreased by 3.2% (*p* < 0.05). The absorption peak, color, and content of Mv-3-O-ara were not affected by HPP alone or during copigmentation with CA, Epi, and GA. In addition, CA had the best effect on the co-coloring of Mv-3-O-gal, while GA was more successful in affecting Mv-3-O-ara during the storage period. Molecular dynamics simulations indicated that the aromatic ring of CA was closest to the A-C plane of Mv-3-O-gal (3.70 Å), resulting in a closer π-π stacking distance and higher bond energy. The favorable impact of GA on Mv-3-O-ara was because the A-C plane aromatic ring of Mv-3-O-ara and the aromatic D ring of GA formed “sandwich” stacking. The results indicated that combining HPP with polyphenols improved color and could be used to process raw materials containing malvidin, such as blueberries.

## 1. Introduction

Anthocyanidins are natural water-soluble plant pigments belonging to flavonoids [1]. The six common anthocyanidins classified by different substitution groups on ring B include delphinidin, cyanidin, petunidin, pelargonidin, peonidin, and malvidin, accounting for about 90% of the known anthocyanidins [2,3]. Anthocyanins exhibit many functional characteristics, such as reducing colon cancer risk by disrupting the cell cycle, reducing proliferation and apoptosis [4], promoting healthy eyes, inducing ciliary muscle relaxation, and contributing to myopia and glaucoma treatment [5].

However, the anthocyanin properties are unstable, limiting its application in food [6,7,8]. Many factors affect the stability of anthocyanins, such as pH [9], the structure of anthocyanins [10], temperature [11], and light [12]. Anthocyanins are more stable at a low pH (acidic conditions), producing red pigments, while a higher pH value yields a blue color [13]. In a buffered solution, the anthocyanin cyanidin-3-O-glucoside is more stable than delphinidin-3-O-glucoside due to the latter’s additional B-5′ hydroxyl group [14]. The increase in hydroxylation and methoxylation reduces the stability of anthocyanins [15]. High temperatures rapidly decrease the anthocyanin level and cause the color to fade over time [16,17], but their overall stability was influenced by a variety of factors. Compared with light conditions, dark storage effectively inhibits anthocyanin degradation, reducing anthocyanin loss [18]. Therefore, improving anthocyanin stability is essential.

Many studies have examined ways to maintain anthocyanidin color stability. Copigmentation is possibly used to stabilize fruit and vegetable color [19]. It combines electron-rich copigmentation π systems with electron-insufficient flavylium cations to form complexes [20]. Intermolecular interaction refers to the process during which anthocyanins associate with non-anthocyanin molecules, such as phenolic acids, metal ions, polyphenols, and polysaccharides, via hydrogen bonding, hydrophobic forces, Van der Waals forces, and ion interactions [21]. In addition, it is also possible for anthocyanins to undergo self-association. Minimal studies are available regarding the impact of different high-pressure processing (HPP) parameters combined with co-colorant reactions on anthocyanin stability [22,23]. Although research has shown that HPP does not negatively impact copigmentation, no relevant articles are available on the combined effect of HPP and co-colorants on anthocyanin storage stability.

Research showed that both pressure and temperature increased the anthocyanin degradation rate [24]. To improve anthocyanin stability, this article explores the effect of HPP on polyphenols and blueberry anthocyanin monomer copigmentation. Beyond that, this article also examined the impact of HPP on the storage stability of two main anthocyanins in blueberries in light and dark conditions after copigmentation with polyphenols. The aim of this study was to provide the feasibility of combining HPP with polyphenol treatment for the processing of raw material containing malvidin and to promote the development of related industries.

## 2. Materials and Methods

### 2.1. Materials

The Mv-3-O-gal and Mv-3-O-ara (purity greater than 98%) were purchased from Baoji Chenguang Biotechnology Co. Ltd. (Baoji, China), while the CA, Epi, and GA were obtained from Chengdu Remifense Biotechnology Co. Ltd. (Chengdu, China). Analytically pure reagents, including disodium hydrogen phosphate and citric acid, were purchased from Chengdu Kelong Chemical Reagent Factory (Chengdu, China), while the chromatographically pure chemicals and reagents, including methanol and formic acid, were acquired from Sigma Company (Saint Louis, MO, USA). Ultrapure water was used throughout the experiment.

### 2.2. Preparation of the Anthocyanin Copigment Solutions

A blueberry juice model solution was prepared using a previously described method with slight modifications [25]. The blueberry juice model buffer was mixed with 6.44 mL of 0.2 mol/L disodium hydrogen phosphate and 13.56 mL of 0.1 mol/L citric acid. The pH was adjusted to 3.6 using disodium hydrogen phosphate or citric acid. Three 10 mmol/L copigment solutions (CA, Epi, and GA) were prepared using the model buffer, while methanol was used to prepare the Mv-3-O-gal and Mv-3-O-ara at 1 mmol/L concentrations. The polyphenol/anthocyanin/model buffer was mixed at a volume ratio of 1:1:3 to obtain a co-color solution at a 1:10 anthocyanin monomer/polyphenol molar ratio [22]. An anthocyanin monomer concentration of 0.2 mmol/L was used in the model solution to avoid self-formation [26]. The mixture was left in the dark for 30 min to reach equilibrium, with all samples set in two parallel positions. The same treatment as the blank control was used, except no polyphenol solution was added.

### 2.3. HPP Treatment

HPP treatment was performed according to a delineated method [25]. A 150 μL sample was placed in a 4 × 6 cm PE food sealing bag, sealed using a heat-sealing machine, and subjected to HPP treatment (HPP 600 MPa/3–5 L, Lineng Ultra High-Pressure Equipment Co. Ltd., Chengdu, China) at 500 MPa/6 min [27]. The treatment process included water as the pressure transfer medium, a linear pressure increased rate of 7.1 MPa/s, pressure fluctuations below 5% during the pressure holding period, and a pressure relief time controlled within 0.5 min.

### 2.4. Color Calculations of the Copigmentation Solutions

The absorbance values at 420, 520, and 620 nm were used to determine the CIELAB parameters for the simulated solutions [28], the temperature of the tested sample was 4 °C. The specific calculation process was as follows [28]:

(1) $$L^{*}=\exp\;(4.611\;-\;0.670\times A_{520})$$

(2) $$a^{*}=\sqrt{-11.666+52.425\times (A_{520})}$$

(3) $$b^{*}=\;-0.711+91.194\times A_{420}\;-\;41.672\times \;A_{520}\;-\;54.220\times A_{620}$$

(4) $$\Delta E=\sqrt{{\left(L^{*}-{L_{0}}^{*}\right)}^{2}+{\left(a^{*}-{a_{0}}^{*}\right)}^{2}+{\left(b^{*}-{b_{0}}^{*}\right)}^{2}}$$

where L* is the brightness of the sample, a* is the redness of the sample, and b* is the yellowness of the sample. The *ΔE* value indicates the degree of color change compared to a sample without added copigments. A value of 0~0.5 represents an insignificant change, 0.5~1.5 signifies a slight change, 1.5~3.0 denotes a noticeable change, 3.0~6.0 represents a clearly visible change, and 6.0~12.0 denotes a substantial change.

### 2.5. Determination of the Maximum Absorption Peak

A 20 μL sample was weighed and scanned using a UV–visible spectrophotometer (UV-1900, Huadelong, Shenzhen, China) at 450–600 nm to obtain the maximum absorption peak. Distilled water was used as a blank [29].

### 2.6. Storage of the Copigmentation Solutions

After HPP, the samples were stored at 4 °C in dark for 105 days (sampling every 15 days) (BCD-155TDGA, Haier, Qingdao, China) and light conditions (LED refrigerator with dedicated light, with a wavelength range of 450–460 nm, intensity of 8500 K, directly shining from the top of the refrigerator) for 20 days (sampling every 5 days) (SC-339J, Haier, Qingdao, China). The maximum absorption peak and anthocyanin content during the storage period were determined.

### 2.7. Determination of the Anthocyanin Content

The anthocyanin content was determined using high-performance liquid chromatography (HPLC, Agilent 1160, Agilent Technologies China Ltd., Beijing, China) according to a method previously described [30]. The copigmentation samples were passed through a 0.45 μm microporous filter membrane for analysis.

The chromatographic conditions included a Kromasil 100-5-C18 column, a column temperature of 30 °C, a column pressure range <400 bar, mobile phase A, consisting of ultrapure water containing 5% formic acid, mobile phase B comprising methanol, a flow rate of 1 mL/min, a detection wavelength of 530 nm, and an injection volume of 20 μL. Gradient elution was as follows: 0 min (90% A, 10% B); 1 min (90% A, 10% B); 15 min (40% A, 60% B); 15.1 min (0% A, 100% B); 18 min (0% A, 100% B); and 20 min (90% A, 10% B).

### 2.8. The Molecular Simulation of the Co-Color Reactions

The molecular dynamics simulations of the polyphenols and anthocyanin co-color solutions were conducted using previously delineated methods [22,31]. ChemDraw 20 was used to plot the initial geometric structures of five exogenous polyphenols, namely CA, Epi, GA, Mv-3-O-gal, and Mv-3-O-ara. The MD simulations were conducted using the GROMACS (version 2019.3 GPU) software, as well as the Amber ff14sb force-field and water TIP3 P models. In a 6 nm cube box, the anthocyanin monomers/copigment/disodium hydrogen phosphate/citric acid/methanol ratio (pH 3.6) was 1:10:26:27:25. The initial simulation system was built using the gmx insert curve command, after which the energy was minimized, followed by 10,000 steps using the steepest descent method, with a minimum constraint value of 1000 KJ/ (moL × nm). After energy minimization, a 100 ps NPT simulation was performed, followed by a 10 ns finished-product simulation. The Vrescale and Parrinello–Rahman algorithms were used for temperature and pressure control, respectively, with a simulation step of 2 fs, a temperature of 300 K, and a pressure of 500 MPa. The electrostatic interaction was determined using the ion mesh (PME) method. A 10 Å Coulomb, electrostatic, and Van der Waals interaction cutoff radius was used, while the hydrogen bond was constrained using the LINCS algorithm.

### 2.9. Statistical Analysis

All tests were conducted in triplicate. Single-factor analysis of variance was used to statistically analyze the absorbance and anthocyanin content, while Tukey’s multiple comparison test was used for post hoc comparison. The data were expressed as mean ± standard deviation, with *p* < 0.05 denoting significant differences. SPSS 21.0 (SPSS Inc., Chicago, IL, USA) was employed for statistical analysis.

## 3. Results and Discussion

### 3.1. The Impact of Different HPP Conditions on Copigmentation Reactions

**Figure 1:**
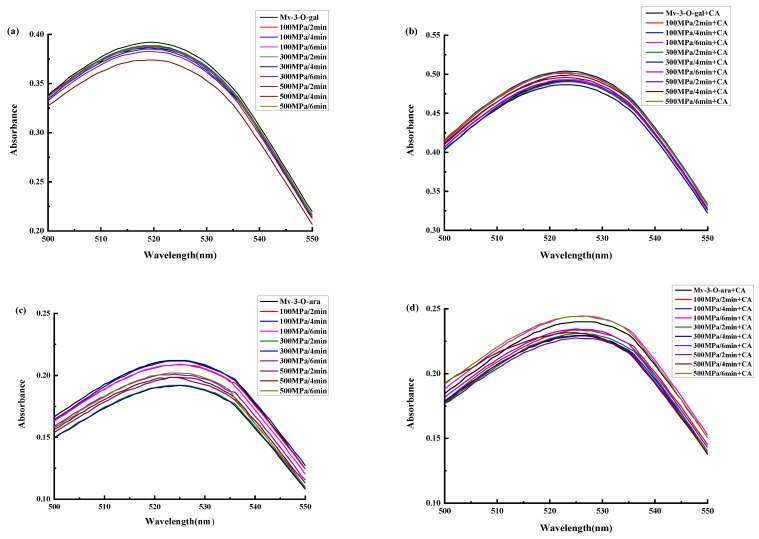
The effect of different HPP conditions on the Mv-3-O-gal and Mv-3-O-ara absorption spectra before and after CA copigmentation. (**a**) Non-supplemented Mv-3-O-gal; (**b**) CA-supplemented Mv-3-O-gal; (**c**) Non-supplemented Mv-3-O-ara; (**d**) CA-supplemented Mv-3-O-ara.

The non-copigmented Mv-3-O-gal and Mv-3-O-ara solutions appeared light red (Table 1 and Table 2). After treatment in different HPP conditions, no significant changes were evident in the maximum absorption wavelengths (Figure 1), absorption peaks, L*, a*, and b* values, and anthocyanin content of the non-copigmented samples (*p* < 0.05). Compared with non-HPP treatment, the *ΔE* values of the Mv-3-O-gal solutions treated at 500 MPa/4 min and 100 MPa/6 min and the Mv-3-O-ara solutions treated at 300 MPa/2 min and 300 MPa/4 min, were between 1.5 and 3, indicating slight color variations. The *ΔE* values of the samples treated in alternative conditions were below 1.5, showing only slight color changes. These results indicated that HPP treatment minimally affected the color of the Mv-3-O-gal and Mv-3-O-ara solutions.

The Mv-3-O-gal and Mv-3-O-ara supplemented with CA solutions appeared deep red. After treatment in different HPP conditions, no significant changes were evident in the maximum absorption wavelengths, absorption peaks, L*, a*, and b* values, and anthocyanin content of the samples supplemented with CA (*p* < 0.05). Compared with the untreated samples, the *ΔE* values of the treated samples were below 1.5, showing no significant or only slight color changes (Table 1 and Table 2). The results indicated that different HPP treatment conditions minimally affected the CA-supplemented Mv-3-O-gal and Mv-3-O-ara, as well as copigmentation.

No significant differences were evident in the impact of different HPP treatment conditions on the copigmentation reaction of malvidin chloride solutions with CA co-coloring. This suggests that HPP treatment at different pressures and durations minimally affected copigmentation.

### 3.2. The Effect of HPP Treatment on Mv-3-O-gal and Mv-3-O-ara Supplemented with CA, Epi, and GA

**Figure 2:**
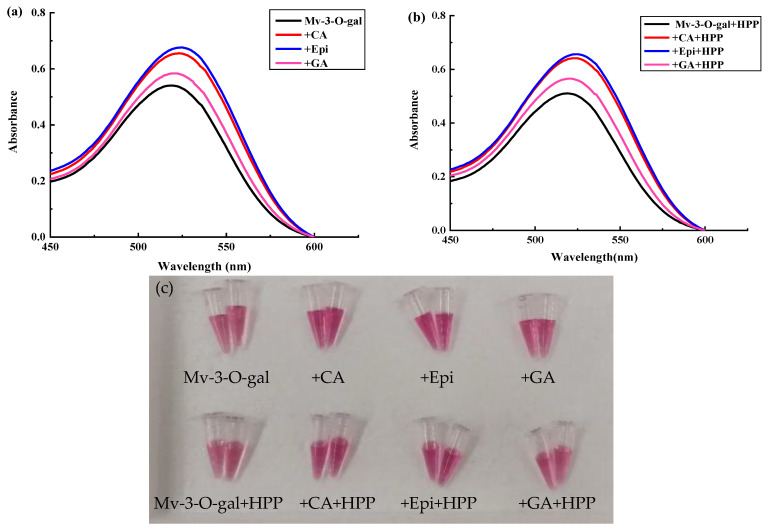
The effect of HPP treatment at 500 MPa/6 min on the absorption spectra (**a**) before HPP, (**b**) after HPP, and (**c**) color of Mv-3-O-gal copigmented with CA, Epi, and GA.

The maximum absorption wavelength (λ_max_) of the non-copigmented Mv-3-O-gal was 518 nm (Figure 2a,b). Mv-3-O-gal supplemented with CA or Epi showed an obvious bathochromic shift. However, no obvious bathochromic shift was evident during Mv-3-O-gal supplemented with GA. Compared with non-copigmented Mv-3-O-gal, the absorption peaks of the Mv-3-O-gal supplemented with CA, Epi, and GA increased by 25%, 28%, and 8.9% (*p* < 0.05), respectively, while the a* values were 16%, 19%, and 6.5% higher (*p* < 0.05) (Table 3). The results showed that CA, Epi, and GA had a significant co-colored effect on Mv-3-O-gal, significantly enhancing its color.

HPP treatment did not significantly affect the λ_max_, peak absorption, L*, a*, and b* values, and anthocyanin content of Mv-3-O-gal alone or supplemented with CA and Epi. The L* and b* values of Mv-3-O-gal supplemented with GA increased by 1.9% and 7.7%, while the a* values decreased by 3.2% (*p* < 0.05) after HPP treatment (Table 3). The results showed that HPP had no significant effect on Mv-3-O-gal supplemented with CA or Epi but reduced its color intensity after being supplemented with GA.

**Figure 3:**
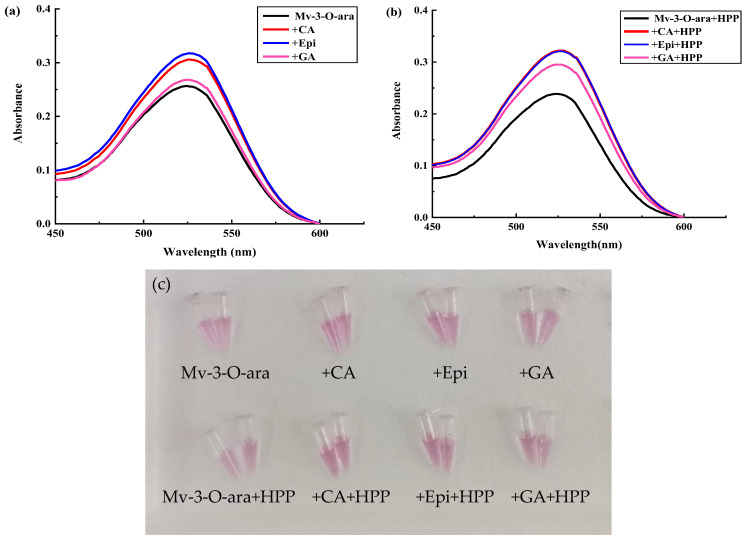
The effect of HPP treatment at 500 MPa/6 min on the absorption spectra (**a**) before HPP, (**b**) after HPP, and (**c**) color of the Mv-3-O-ara copigmented with CA, Epi, and GA.

Compared with non-copigmented Mv-3-O-ara, or samples supplemented with CA, Epi and GA did not show a significant bathochromic shift (Figure 3a,b). The absorption peaks of Mv-3-O-ara supplemented with CA and Epi increased by 24%, while the other parameters displayed no significant changes (Table 4). The results showed that HPP did not significantly affect the Mv-3-O-ara supplemented with CA, Epi, and GA.

The results showed that HPP treatment did not influence the anthocyanin content, which was consistent with previous studies [32,33,34,35]. Furthermore, HPP did not adversely affect polyphenols and anthocyanin copigmentation. Zou et al. [22] discovered that HPP treatment at 500 MPa for 30 min could enhance the rate of copigmentation reactions between pelargonidin-3-glucoside and catechin. However, He et al. [36] indicated a reduction in the absorption peak by 11.65% for copigmented *Vitis amurensis* anthocyanins stored at 20 °C for 10 days after HPP treatment at 300 MPa for 3 min. Non-covalent bonds, such as hydrogen, hydrophobic, and ionic bonds, may be the cause of differing anthocyanin monomer and polyphenols interaction results after HPP treatment [37].In these conditions, HPP did not influence the co-colored reaction, which benefitted product processing. Therefore, CA, Epi, and GA can be added during HPP to enhance product color and stability.

### 3.3. The Effect of HPP on the Anthocyanin Solution Color During Storage

Figure S1 shows the color of Mv-3-O-gal and Mv-3-O-ara in light and dark conditions after polyphenol copigmentation and HPP treatment. As the storage time was extended, the color of the two anthocyanin solutions alone faded gradually, which occurred faster in light than in dark storage conditions. However, adding CA, Epi, and GA slowed the fading to varying degrees in both storage conditions (Figure S1). Compared with Epi and GA, CA had a better copigmentation effect on Mv-3-O-gal, while GA was most suitable for Mv-3-O-ara (Figure S1c,d).

**Figure 4:**
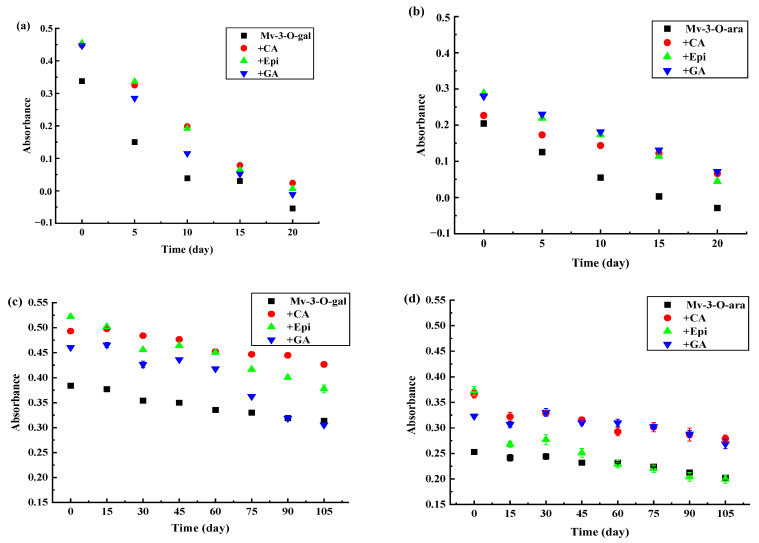
The absorbance changes in the Mv-3-O-gal and Mv-3-O-ara solutions supplemented with polyphenols after HPP treatment during storage at 4 °C. (**a**) Mv-3-O-gal stored under light condition; (**b**) Mv-3-O-ara stored under light condition; (**c**) Mv-3-O-gal under dark condition; (**d**) Mv-3-O-ara stored under dark condition.

Figure 4 shows the maximum absorption of all the samples in light and dark conditions at 450–600 nm. In light conditions, the Mv-3-O-gal and Mv-3-O-ara solutions without copigmentation faded significantly with the prolongation of storage time. (*p* < 0.05). Previous research showed that Mv-3-O-gal faded completely after storage at room temperature in light conditions for 55 d, which was roughly consistent with the results of this study [38]. CA, Epi, and GA supplements delayed the decline in absorption peaks of the Mv-3-O-gal solution compared to the samples not exposed to copigmentation. The absorption peaks of the samples supplemented with CA, Epi, and GA copigmentation increased by 80%, 79%, and 66% at 10 d compared to the untreated samples. The co-coloring effect of CA exceeded that of Epi and GA. The absorbance decreased during the storage period; at 20 d, only CA still exhibited a copigmentation impact on Mv-3-O-gal. A similar result was evident for Mv-3-O-ara (Figure 4b).

In dark storage conditions, the light absorption peaks of all the samples decreased gradually at 450–600 nm (*p* < 0.05). The Mv-3-O-gal solution displayed the best copigmentation effect at 45 d (Figure 4c). Compared with the uncolored sample, the absorption peak of the Mv-3-O-gal solution increased by 26%, 24%, and 19% after adding CA, Epi, and GA compared to the uncolored sample. The three agents exhibited relatively stable copigmentation effects, while CA was consistently more successful in Mv-3-O-gal, which was similar to Mv-3-O-ara copigmentation. The absorption peaks of the Mv-3-O-ara solution increased by 26%, 7.5%, and 25% at 60 d compared with the uncolored sample and by 27%, −1.5%, and 24% at 105 d (Figure 4d).

The results indicated that CA and GA improved the storage stability of both Mv-3-O-gal and Mv-3-O-ara after HPP treatment. Similarly, Kopjar et al. [39] showed that CA copigmentation improved the anthocyanin stability in blackberry juice during storage at 4 °C for 30 d. Ning et al. [40] found that GA was the most effective for anthocyanin copigmentation in blueberry juice exposed to HPP at 550 MPa/10 min/25 °C. Therefore, GA stabilized anthocyanin construction in blueberry juice. CA can effectively achieve copigmentation, possibly since it was the main free phenolic acid in blueberries, with a content of up to 200–1000 mg/kg, which was suitable for binding with blueberry anthocyanins [41,42]. However, co-color degradation may still occur due to the breakage caused by co-coloring reactions during storage [43].

### 3.4. The Effect of HPP on the Anthocyanidin Content

**Figure 5:**
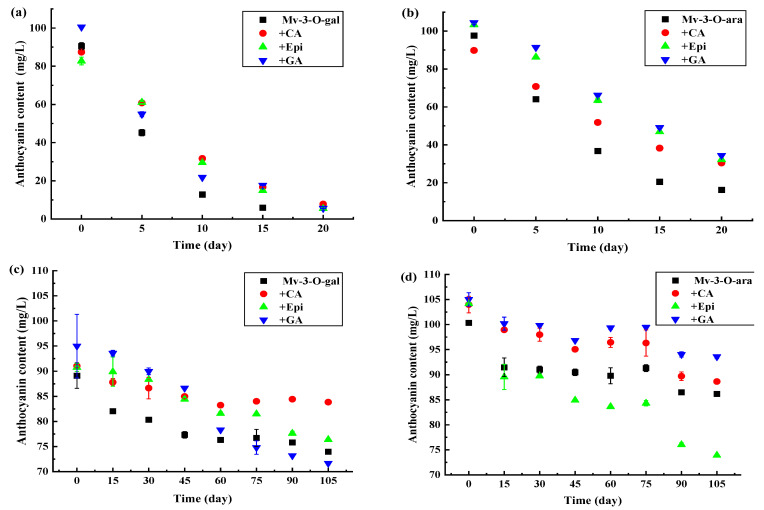
The anthocyanin content changes in the Mv-3-O-gal and Mv-3-O-ara solutions supplemented with polyphenols after HPP treatment during storage at 4 °C. (**a**) Mv-3-O-gal stored under light condition; (**b**) Mv-3-O-ara stored under light condition; (**c**) Mv-3-O-gal under dark condition; (**d**) Mv-3-O-ara stored under dark condition.

As the storage period was extended, the anthocyanin levels decreased significantly (*p* < 0.05) in the uncolored Mv-3-O-gal and Mv-3-O-ara during 4 °C light storage conditions (Figure 5a,b) while adding CA, Epi, and GA substantially delayed anthocyanin degradation. At 10 d, the anthocyanin content in the Mv-3-O-gal supplemented with CA, Epi, and GA was 59%, 56%, and 41% higher than in the uncolored samples (Figure 5a). Similar results were evident for the Mv-3-O-ara solution copigmentation (Figure 5b). At 15 d, the anthocyanin content in Mv-3-O-ara supplemented with CA, Epi, and GA was 46%, 56%, and 58% higher than in the uncolored samples. The results showed that the anthocyanin content variation was consistent with the maximum absorbance changes during storage in light conditions.

Although slow anthocyanin degradation was evident in all the samples in dark storage conditions at 4 °C, the Mv-3-O-gal and Mv-3-O-ara solutions displayed anthocyanin content variation after copigmentation (Figure 5c,d). At 30 d, the anthocyanin levels in the Mv-3-O-gal supplemented with CA, Epi, and GA were 7.2%, 9.0%, and 10% higher than the uncolored samples. After 60 d, anthocyanin content degradation accelerated in the Mv-3-O-gal solution supplemented with GA, deteriorating faster than the uncolored samples. However, the anthocyanin levels in the Mv-3-O-gal solution supplemented with CA and Epi remained higher than in the uncolored sample (Figure 5a). Although the anthocyanin content in the Mv-3-O-gal solution supplemented with GA was lower than in the uncolored sample at 75 d, no significant color changes were evident (Figure S1c), indicating that the decrease in anthocyanin content was faster than color fading.

Hager et al. [44] found that although the anthocyanin content of berries canned in water decreased linearly, the percent polymeric color increased during 60 d of storage at 25 °C, which could be due to extensive anthocyanin polymerization during storage [45]. Muche et al. [46] studied the anthocyanin content and color changes in different grape varieties stored at various temperatures, and their results showed that the anthocyanin content decreased in all the samples while the polymetric color increased, indicating that the color and content changes might not occur simultaneously. The different results may be related to anthocyanin, copigment, treatment conditions, and molar ratio variation between the anthocyanins and copigments.

### 3.5. Mechanism Study on the Influence of HPP on Co-Colored Reaction Based on Molecular Simulation

**Figure 6:**
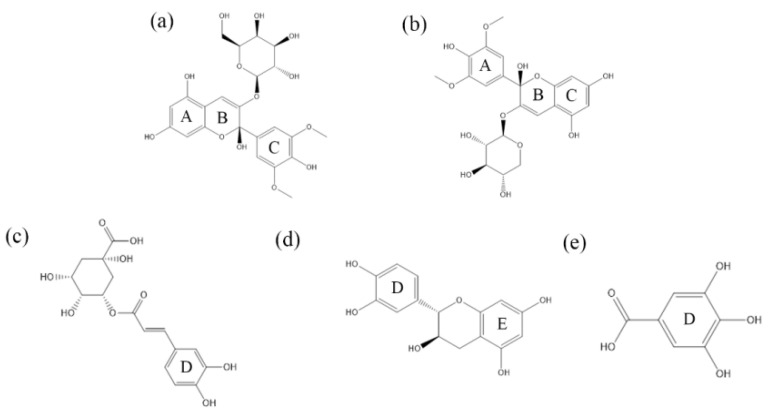
The anthocyanin monomer and polyphenol molecular structure formula. (**a**) Mv-3-O-gal, (**b**) Mv-3-O-ara, (**c**) CA, (**d**) Epi, and (**e**) GA.

The GROMACS software (version 2019.3 GPU) was used for the molecular dynamics simulation to clarify the co-color reaction that combines malvidin pigment anthocyanin monomers with CA, Epi, and GA. Figure 6 shows the molecular structure formula of each substance.

**Figure 7:**
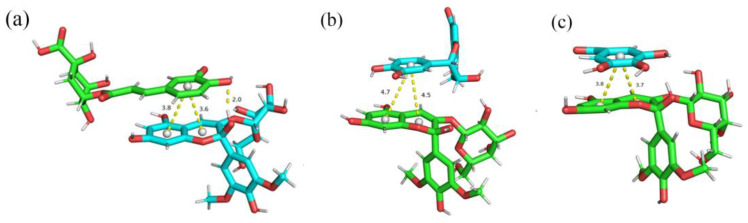
The interaction between malvidin-3-O-galactoside and polyphenols by molecular simulation. (**a**) Mv-3-O-gal and CA; (**b**) Mv-3-O-gal and Epi; (**c**) Mv-3-O-gal and GA.

Figure 7 shows a molecular simulation diagram of the interaction between Mv-3-O-gal and CA, Epi, and GA. The average minimum distance from the A-C plane of Mv-3-O-gal to the polyphenols D ring was ordered as CA < GA < Epi, while that of the hydrogen bonds was ordered as Epi < CA < GA (Table 5). The closer hydrogen bond distance between Mv-3-O-gal and Epi may be due to the presence of five hydroxyl groups, which can form hydrogen bonds with monomers. However, although Epi has two aromatic rings, its configuration was not easily twisted, resulting in a considerable π-π stacking distance between the Epi B ring and anthocyanin monomer D ring. Furthermore, π-π stacking was caused by the hydrophobic interaction between co-colorant molecules and anthocyanin molecular rings [47]. The relatively close π-π stacking distance between Mv-3-O-gal and CA, GA may be because the simple aromatic ring in the A-C plane of Mv-3-O-gal was relatively conjugated with 10 electrons (the methanol base form cannot be completely conjugated), which was conducive to π-π stacking formation. Hydrophobic bonding and π-π stacking have been clearly identified as driving forces for copigmentation [25]. CA exhibited the best co-coloring effect on Mv-3-O-gal in both light and dark storage conditions at 4 °C due to the shortest distance between its aromatic D-ring and the A-C plane of Mv-3-O-gal, the highest hydrogen bonding, and stronger binding ability. Similarly, Chen et al. [37] found that CA and cyanidin-3-O-glucoside formed four hydrogen bonds and π-π stacking forces, resulting in a relatively stable system.

**Figure 8:**
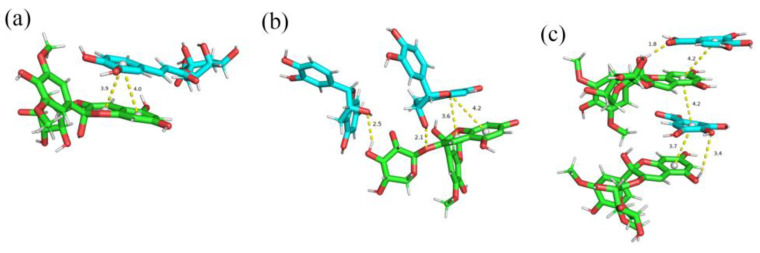
The interaction between malvidin-3-O-arabinoside and polyphenols by molecular simulation. (**a**) Mv-3-O-ara and CA; (**b**) Mv-3-O-ara and Epi; (**c**) Mv-3-O-ara and GA.

The closest π-π stacking distance between Mv-3-O-ara and GA may be because the simple A-C plane aromatic ring of Mv-3-O-ara and the aromatic D ring of GA form “sandwich” stacking (Figure 8c), making the rings more compact. Although the distance between Epi and CA was also relatively close, it did not form a “sandwich” (Figure 8a,b). Kunsági-Máté et al. [48] showed that anthocyanins and polyphenols require lower activation energies during the formation of “sandwich” structures and form more stable complexes. The results indicated that GA was more conducive to Mv-3-O-ara stability due to the “sandwich” stacking.

## 4. Conclusions

This study found that the change in HPP pressure and time had no significant effect on the copigmentation. No substantial impact was evident on the copigmentation of the Mv-3-O-gal and Mv-3-O-ara with CA, Epi, and GA after HPP treatment. Light more significantly affected anthocyanin fading and degradation than dark storage conditions, while polyphenol addition can alleviate this impact. CA and GA significantly affected the anthocyanin color and content in Mv-3-O-gal and Mv-3-O-ara in both dark and light conditions. Direct molecular simulation suggested that CA and GA display a better copigmentation effect on Mv-3-O-gal and Mv-3-O-ara. Therefore, CA and GA combined with HPP might be used in some juices processing industries to improve Mv-3-O-gal and Mv-3-O-ara storage stability. However, this study only explored the copigmentation reaction between two anthocyanin monomers and polyphenols in a model system. Further research is necessary to explore the actual copigmentation effect of CA and GA in some real food systems.

## Figures and Tables

**Table 1:** The effect of HPP on Mv-3-O-gal before and after CA copigmentation in different conditions.

Different Conditions	Absorption Peak	Color				Anthocyanin Content (mg/L)
		L*	a*	b*	*ΔE*	
Mv-3-O-gal	0.39 ± 0.01 ^b^	78.05 ± 0.11 ^a^	20.59 ± 0.09 ^b^	−5.23 ± 0.12 ^a^	-	98.49 ± 0.18 ^ab^
100 MPa/2 min	0.39 ± 0.00 ^b^	78.42 ± 0.33 ^a^	20.29 ± 0.27 ^b^	−4.73 ± 0.05 ^a^	0.73 ± 0.25 ^a^	93.96 ± 2.66 ^b^
100 MPa/4 min	0.39 ± 0.02 ^b^	78.66 ± 0.00 ^a^	20.09 ± 0.00 ^b^	−4.74 ± 0.35 ^a^	0.94 ± 0.18 ^a^	94.12 ± 2.92 ^b^
100 MPa/6 min	0.39 ± 0.01 ^b^	78.42 ± 0.26 ^a^	20.29 ± 0.21 ^b^	−4.93 ± 0.24 ^a^	0.56 ± 0.41 ^a^	99.46 ± 1.27 ^ab^
300 MPa/2 min	0.39 ± 0.01 ^b^	78.21 ± 0.33 ^a^	20.46 ± 0.27 ^b^	−4.90 ± 0.11 ^a^	0.46 ± 0.27 ^a^	96.76 ± 1.95 ^ab^
300 MPa/4 min	0.39 ± 0.02 ^b^	78.45 ± 0.15 ^a^	20.27 ± 0.12 ^b^	−4.68 ± 0.12 ^a^	0.75 ± 0.22 ^a^	94.14 ± 3.97 ^bc^
300 MPa/6 min	0.39 ± 0.00 ^b^	78.32 ± 0.11 ^a^	20.37 ± 0.09 ^b^	−4.82 ± 0.01 ^a^	0.44 ± 0.23 ^a^	97.79 ± 0.84 ^ab^
500 MPa/2 min	0.38 ± 0.01 ^b^	78.55 ± 0.15 ^a^	20.18 ± 0.12 ^b^	−4.61 ± 0.14 ^a^	0.91 ± 0.04 ^a^	97.06 ± 0.76 ^ab^
500 MPa/4 min	0.37 ± 0.01 ^b^	79.08 ± 0.37 ^a^	19.75 ± 0.31 ^b^	−4.25 ± 0.50 ^a^	1.65 ± 0.69 ^a^	98.61 ± 0.92 ^ab^
500 MPa/6 min	0.39 ± 0.01 ^b^	78.34 ± 0.15 ^a^	20.35 ± 0.12 ^a^	−4.68 ± 0.51 ^a^	0.68 ± 0.02 ^a^	93.88 ± 2.07 ^b^
Mv-3-O-gal + CA	0.49 ± 0.01 ^a^	72.00 ± 0.14 ^b^	25.37 ± 0.10 ^a^	−7.69 ± 0.09 ^b^	-	101.92 ± 3.21 ^ab^
100 MPa/2 min + CA	0.50 ± 0.02 ^a^	71.47 ± 0.47 ^b^	25.77 ± 0.36 ^a^	−7.58 ± 0.31 ^b^	0.75 ± 0.49 ^a^	100.23 ± 2.41 ^ab^
100 MPa/4 min + CA	0.50 ± 0.02 ^a^	71.40 ± 0.03 ^b^	25.83 ± 0.03 ^a^	−7.55 ± 0.00 ^b^	0.77 ± 0.04 ^a^	101.61 ± 2.42 ^ab^
100 MPa/6 min + CA	0.49 ± 0.01 ^a^	71.95 ± 0.95 ^b^	25.40 ± 0.73 ^a^	−7.27 ± 0.20 ^b^	0.96 ± 0.02 ^a^	99.33 ± 0.58 ^ab^
300 MPa/2 min + CA	0.49 ± 0.02 ^a^	72.07 ± 0.72 ^b^	25.31 ± 0.55 ^a^	−7.45 ± 0.68 ^b^	0.81 ± 0.30 ^a^	100.36 ± 0.12 ^ab^
300 MPa/4 min + CA	0.49 ± 0.01 ^a^	72.34 ± 0.48 ^b^	25.11 ± 0.37 ^a^	−6.95 ± 0.66 ^b^	0.87 ± 0.85 ^a^	102.79 ± 1.56 ^a^
300 MPa/6 min + CA	0.49 ± 0.01 ^a^	72.00 ± 0.20 ^b^	25.37 ± 0.16 ^a^	−7.25 ± 0.26 ^b^	0.48 ± 0.24 ^a^	101.73 ± 2.63 ^ab^
500 MPa/2 min + CA	0.50 ± 0.00 ^a^	71.93 ± 0.10 ^b^	25.42 ± 0.08 ^a^	−7.05 ± 0.02 ^b^	0.65 ± 0.01 ^a^	101.78 ± 0.87 ^ab^
500 MPa/4 min + CA	0.50 ± 0.01 ^a^	71.59 ± 0.44 ^b^	25.68 ± 0.34 ^a^	−7.39 ± 0.52 ^b^	0.79 ± 0.17 ^a^	99.75 ± 0.66 ^ab^
500 MPa/6 min + CA	0.50 ± 0.01 ^a^	71.50 ± 0.37 ^b^	25.75 ± 0.29 ^a^	−7.06 ± 0.08 ^b^	0.94 ± 0.27 ^a^	98.95 ± 1.76 ^ab^

Note: The *ΔE* values of both the non-toned and toned samples are derived by calculating the differences from their respective values before HPP treatment. Different letters, such as ‘a’ and ‘b’, in the same column indicate significant differences.

**Table 2:** The effect of HPP on Mv-3-O-ara before and after CA copigmentation in different conditions.

Different Conditions	Absorption Peak	Color				Anthocyanin Content (mg/L)
		L*	a*	b*	*ΔE*	
Mv-3-O-ara	0.21 ± 0.01 ^abcde^	87.38 ± 0.33 ^abcd^	12.36 ± 0.32 ^abcd^	−2.96 ± 0.39 ^a^	-	75.92 ± 1.16 ^a^
100 MPa/2 min	0.21 ± 0.01 ^bcde^	87.62 ± 0.17 ^abcd^	12.13 ± 0.16 ^abcd^	−2.70 ± 0.50 ^a^	0.57 ± 0.09 ^a^	73.47 ± 1.61 ^ab^
100 MPa/4 min	0.21 ± 0.02 ^abcde^	87.44 ± 0.17 ^abcd^	12.30 ± 0.16 ^abcd^	−2.96 ± 0.26 ^a^	0.25 ± 0.07 ^a^	72.16 ± 0.80 ^ab^
100 MPa/6 min	0.21 ± 0.01 ^bcde^	87.59 ± 0.04 ^abcd^	12.16 ± 0.04 ^abcd^	−3.06 ± 0.06 ^a^	0.30 ± 0.07 ^a^	73.76 ± 0.00 ^ab^
300 MPa/2 min	0.19 ± 0.01 ^e^	88.56 ± 0.42 ^a^	11.18 ± 0.43 ^d^	−2.84 ± 0.22 ^a^	1.68 ± 0.58 ^a^	72.93 ± 1.03 ^ab^
300 MPa/4 min	0.19 ± 0.00 ^e^	88.56 ± 0.76 ^a^	11.18 ± 0.77 ^d^	−2.73 ± 0.01 ^a^	1.69 ± 1.07 ^a^	74.81 ± 1.62 ^ab^
300 MPa/6 min	0.20 ± 0.01 ^cde^	88.00 ± 0.29 ^abc^	11.75 ± 0.29 ^bcd^	−2.53 ± 0.23 ^a^	1.01 ± 0.25 ^a^	73.99 ± 0.61 ^ab^
500 MPa/2 min	0.20 ± 0.01 ^de^	88.24 ± 0.13 ^ab^	11.51 ± 0.13 ^cd^	−2.62 ± 0.36 ^a^	1.28 ± 0.07 ^a^	74.48 ± 0.09 ^ab^
500 MPa/4 min	0.20 ± 0.01 ^de^	88.15 ± 0.92 ^abc^	11.59 ± 0.92 ^bcd^	−2.58 ± 0.21 ^a^	1.17 ± 1.26 ^a^	73.46 ± 0.97 ^ab^
500 MPa/6 min	0.20 ± 0.01 ^cde^	87.97 ± 0.75 ^abc^	11.77 ± 0.75 ^bcd^	−2.95 ± 0.32 ^a^	0.91 ± 0.97 ^a^	74.93 ± 0.20 ^ab^
Mv-3-O-ara + CA	0.24 ± 0.00 ^ab^	85.87 ± 0.33 ^d^	13.80 ± 0.31 ^a^	−3.43 ± 0.20 ^a^	-	73.00 ± 0.24 ^ab^
100 MPa/2 min + CA	0.23 ± 0.02 ^abcd^	86.28 ± 0.25 ^bcd^	13.42 ± 0.23 ^abc^	−3.32 ± 0.27 ^a^	0.62 ± 0.26 ^a^	72.86 ± 0.40 ^ab^
100 MPa/4 min + CA	0.23 ± 0.01 ^abcd^	86.45 ± 0.90 ^bcd^	13.25 ± 0.86 ^abc^	−3.00 ± 0.02 ^a^	1.08 ± 0.93 ^a^	72.74 ± 0.31 ^ab^
100 MPa/6 min + CA	0.24 ± 0.02 ^a^	85.67 ± 0.12 ^d^	13.99 ± 0.11 ^a^	−3.25 ± 0.13 ^a^	0.33 ± 0.21 ^a^	72.86 ± 0.82 ^ab^
300 MPa/2 min + CA	0.23 ± 0.01 ^abcd^	86.39 ± 0.16 ^bcd^	13.31 ± 0.16 ^abc^	−3.28 ± 0.16 ^a^	0.74 ± 0.18 ^a^	72.84 ± 0.84 ^ab^
300 MPa/4 min + CA	0.23 ± 0.01 ^abcd^	86.51 ± 0.25 ^bcd^	13.20 ± 0.23 ^abc^	−2.52 ± 0.19 ^a^	1.26 ± 0.37 ^a^	72.77 ± 1.37 ^ab^
300 MPa/6 min + CA	0.23 ± 0.01 ^abc^	86.16 ± 0.08 ^cd^	13.53 ± 0.08 ^ab^	−2.75 ± 0.05 ^a^	0.79 ± 0.10 ^a^	72.83 ± 0.58 ^ab^
500 MPa/2 min + CA	0.23 ± 0.02 ^abcd^	86.60 ± 0.86 ^abcd^	13.11 ± 0.82 ^abcd^	−3.00 ± 1.16 ^a^	1.32 ± 1.29 ^a^	71.24 ± 2.13 ^b^
500 MPa/4 min + CA	0.23 ± 0.00 ^abcd^	86.25 ± 0.45 ^bcd^	13.45 ± 0.43 ^abc^	−3.09 ± 0.23 ^a^	0.64 ± 0.63 ^a^	73.55 ± 0.64 ^ab^
500 MPa/6 min + CA	0.24 ± 0.01 ^a^	85.62 ± 0.69 ^d^	14.304 ± 0.64 ^a^	−2.94 ± 0.68 ^a^	0.86 ± 0.78 ^a^	74.63 ± 0.40 ^ab^

Note: The *ΔE* values of both the non-toned and toned samples are derived by calculating the differences from their respective values before HPP treatment. Different letters, such as ‘a’ and ‘b’, in the same column indicate significant differences.

**Table 3:** The effect of HPP treatment at 500 MPa/6 min on Mv-3-O-gal supplemented with CA, Epi, and GA.

Polyphenol Copigmentation	Peak Absorption	Color			Anthocyanin Content (mg/L)
		L*	a*	b*	
Mv-3-O-gal	0.56 ± 0.00 ^d^	69.26 ± 0.07 ^bc^	27.46 ± 0.05 ^de^	−7.64 ± 0.32 ^a^	93.33 ± 4.62 ^ab^
+CA	0.70 ± 0.00 ^ab^	63.03 ± 0.03 ^ef^	32.12 ± 0.02 ^ab^	−11.02 ± 0.05 ^d^	96.36 ± 1.16 ^a^
+Epi	0.72 ± 0.02 ^a^	62.18 ± 0.71 ^f^	32.75 ± 0.52 ^a^	−11.22 ± 0.32 ^d^	96.70 ± 1.27 ^a^
+GA	0.61 ± 0.00 ^c^	66.88 ± 0.06 ^d^	29.25 ± 0.05 ^c^	−9.15 ± 0.01 ^c^	90.17 ± 0.46 ^ab^
+HPP	0.55 ± 0.00 ^de^	70.02 ± 0.17 ^b^	27.12 ± 0.13 ^ef^	−7.42 ± 0.02 ^a^	90.54 ± 0.02 ^ab^
+CA + HPP	0.68 ± 0.00 ^b^	63.78 ± 0.06 ^e^	31.56 ± 0.04 ^b^	−10.75 ± 0.04 ^d^	93.82 ± 1.38 ^ab^
+Epi + HPP	0.70 ± 0.01 ^ab^	63.27 ± 0.54 ^ef^	31.94 ± 0.40 ^ab^	−11.00 ± 0.35 ^d^	94.15 ± 0.47 ^ab^
+GA + HPP	0.58 ± 0.01 ^cd^	68.15 ± 0.52 ^c^	28.29 ± 0.39 ^d^	−8.44 ± 0.19 ^b^	87.08 ± 1.29 ^b^

Note: Different letters indicate significant differences in the same column (*p* < 0.05).

**Table 4:** The effect of HPP treatment at 500 MPa/6 min on the Mv-3-O-ara supplemented with CA, Epi, and GA.

Polyphenol Copigmentation	Peak Absorption	Color			Anthocyanin Content (mg/L)
		L*	a*	b*	
Mv-3-O-ara	0.25 ± 0.01 ^bc^	85.33 ± 0.77 ^abc^	14.30 ± 0.071 ^ab^	−3.60 ± 0.40 ^abcd^	88.75 ± 2.05 ^ab^
+CA	0.31 ± 0.03 ^a^	82.29 ± 2.42 ^c^	17.01 ± 2.10 ^a^	−4.80 ± 0.44 ^de^	102.16 ± 2.74 ^a^
+Epi	0.31 ± 0.02 ^a^	81.89 ± 1.01 ^c^	17.37 ± 0.87 ^a^	−5.08 ± 0.64 ^e^	91.79 ± 9.86 ^ab^
+GA	0.26 ± 0.01 ^abc^	84.87 ± 0.76 ^abc^	14.72 ± 0.70 ^ab^	−4.40 ± 0.20 ^bcde^	89.27 ± 2.23 ^ab^
+HPP	0.22 ± 0.02 ^c^	86.92 ± 1.24 ^ab^	12.80 ± 1.19 ^b^	−3.42 ± 0.36 ^abc^	80.38 ± 9.13 ^ab^
+CA + HPP	0.32 ± 0.02 ^a^	81.64 ± 0.97 ^c^	17.59 ± 0.83 ^a^	−4.61 ± 0.05 ^cde^	96.15 ± 2.04 ^a^
+Epi + HPP	0.31 ± 0.01 ^a^	81.78 ± 0.70 ^c^	17.47 ± 0.60 ^a^	−4.87 ± 0.03 ^de^	95.30 ± 0.68 ^a^
+GA + HPP	0.29 ± 0.00 ^ab^	83.02 ± 0.04 ^bc^	16.39 ± 0.03 ^a^	−3.81 ± 0.36 ^abcde^	93.77 ± 4.46 ^ab^

Note: Different letters indicate significant differences in the same column (*p* < 0.05).

**Table 5:** The average minimum distance between Mv-3-O-gal/Mv-3-O-ara and polyphenols.

Anthocyanin	Average Minimum Distance (Å)				
	Copigmentation	AC-Plane to D-Ring	B-Ring to D-Ring	AC-Plane to E-Ring	Average Minimum Distance of Hydrogen Bonds
Mv-3-O-gal	CA	3.70	3.80	-	2.14
	Epi	4.60	5.00	3.93	1.80
	GA	3.75	3.68	-	2.17
Mv-3-O-ara	CA	3.95	3.60	-	3.00
	Epi	3.75	3.90	3.90	2.18
	GA	3.62	3.85	-	1.93

## Data Availability

The original contributions presented in this study are included in the article/Supplementary Material, further inquiries can be directed to the corresponding author.

## Supplementary Materials

The following supporting information can be downloaded at: https://www.mdpi.com/article/10.3390/foods13233756/s1, Figure S1. The color changes in Mv-3-O-gal and Mv-3-O-ara solutions supplemented with polyphenols after HPP treatment during storage at 4 °C.
