# Supplementary material for: The Effect of High-Pressure Processing on the Copigmentation and Storage Stability of Polyphenols with Anthocyanin Monomers
Source: Foods. 2024 Nov 23;13(23):3756. doi: 10.3390/foods13233756 (PMC11639931; doi:10.3390/foods13233756)
Supplement: Supplementary file 1 [file foods-13-03756-s001.zip › foods-3316343-supplementary.pdf]

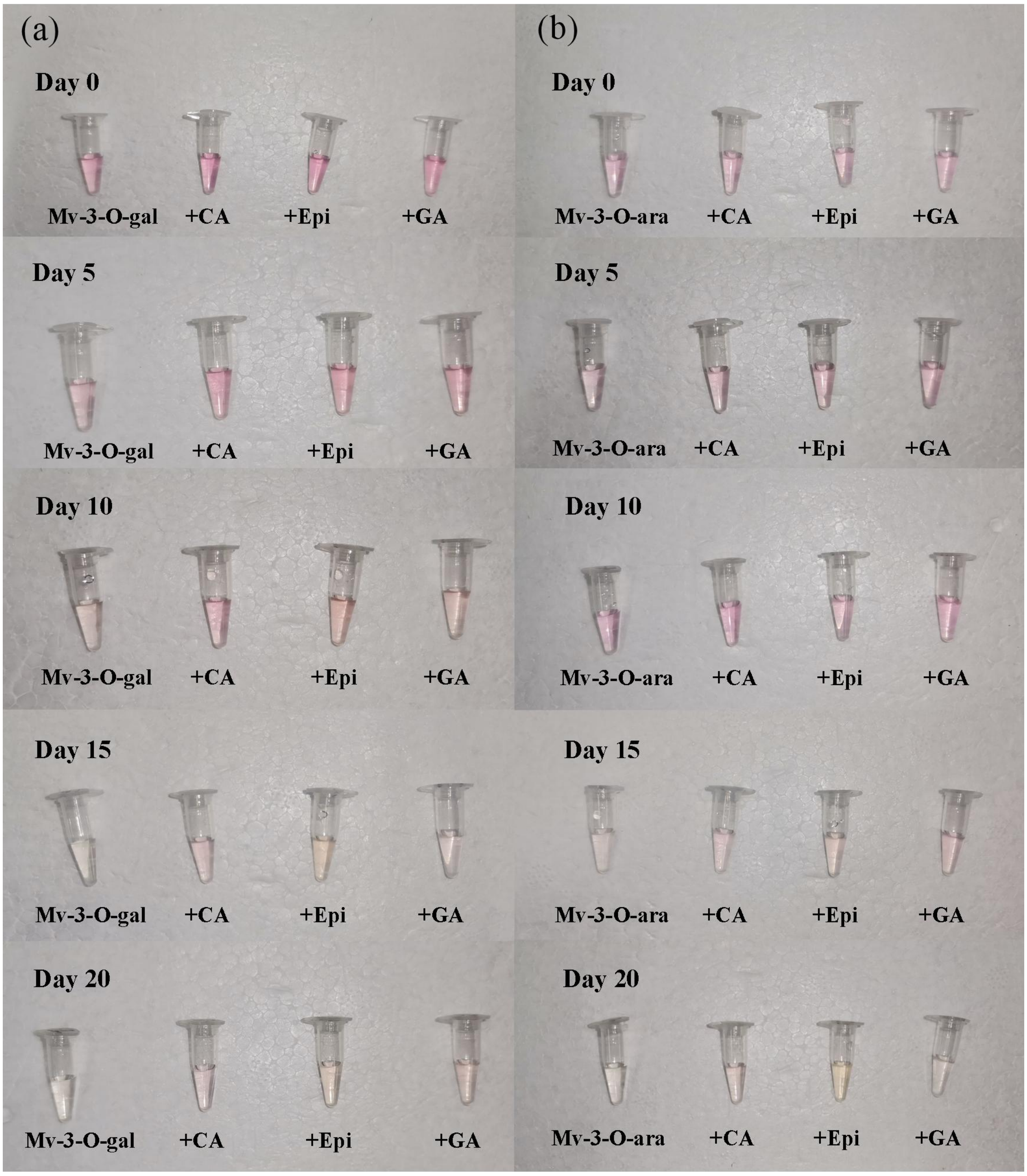
(a)
Day 0
Mv-3-O-gal
+CA
+Epi
+GA
Day 5
Mv-3-O-gal
+CA
+Epi
+GA
Day 10
Mv-3-O-gal
+CA
+Epi
+GA
Day 15
Mv-3-O-gal
+CA
+Epi
+GA
Day 20
Mv-3-O-gal
+CA
+Epi
+GA
(b)
Day 0
Mv-3-O-ara
+CA
+Epi
+GA
Day 5
Mv-3-O-ara
+CA
+Epi
+GA
Day 10
Mv-3-O-ara
+CA
+Epi
+GA
Day 15
Mv-3-O-ara
+CA
+Epi
+GA
Day 20
Mv-3-O-ara
+CA
+Epi
+GA

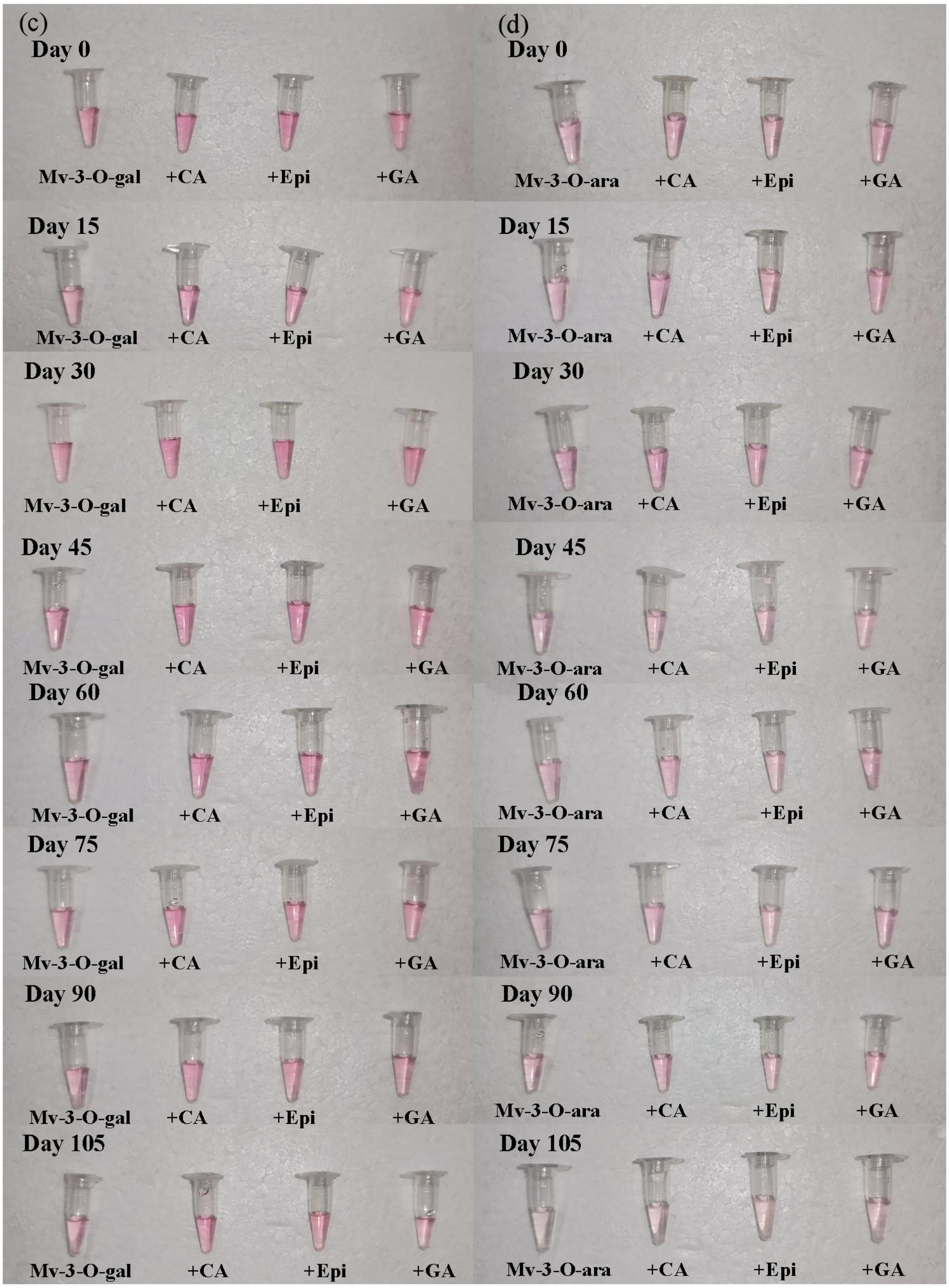

Figure S1. The color changes in Mv-3-O-gal and Mv-3-O-ara solutions supplemented with polyphenols after HPP treatment during storage at 4°C.

(a)Mv-3-O-gal stored under light condition; (b) Mv-3-O-ara stored under light condition;
(c)Mv-3-O-gal under dark condition; (d) Mv-3-O-ara stored under dark condition
